# Computational Fluid Dynamic Evaluation of Deep Inferior Epigastric Artery Perforator (DIEP) Flap End-to-Side Anastomosis

**DOI:** 10.7759/cureus.24650

**Published:** 2022-05-01

**Authors:** Sanjay K Jinka, Ashoka G Jinka, Jeffrey E Janis

**Affiliations:** 1 Medicine, Northeast Ohio Medical University, Rootstown, USA; 2 Computational Modeling, Independent Consultant, Maumee, USA; 3 Plastic and Reconstructive Surgery, The Ohio State University Wexner Medical Center, Columbus, USA

**Keywords:** end-to-side anastomosis, diep flap, free flap, simulation, ischemia, computational fluid dynamics

## Abstract

Background

End-to-side (ETS) microvascular anastomoses are utilized within deep inferior epigastric artery perforator (DIEP) flap breast reconstruction procedures. Optimization of these anastomoses via a computational fluid dynamic (CFD) model can minimize ischemia and contribute to flap success.

Methods

A CFD model of a deep inferior epigastric artery to internal mammary artery anastomosis was constructed with OpenFOAM software (OpenCFD Ltd., Bracknell, UK). Blood was modelled as an incompressible Newtonian fluid. Viscosity and density were assumed to be constant throughout the simulation. Mean arterial pressure was held constant at 100 mmHg. Individual virtual meshes were created for 30-, 45-, 60-, 75-, and 90-degree anastomotic angle simulations. Fluid flow was visualized with line integral convolution (LIC) and pure fluid velocity (PFV) techniques. Vessel wall shear stress (WSS) was also visualized.

Results

The LIC revealed blood recirculation was associated with large anastomotic angles with minimal to no recirculation seen in the 45- and 30-degree simulations. Any recirculation visualized was confined to the toe of the bifurcation. This recirculation was associated with stagnation in the toe of the graft vessel as well, as visualized by the PFV model. A linear relationship was identified between anastomotic angle and percentage of stagnant fluid, with stagnation increasing as the anastomotic angle increased. Wall shear stress increased with the anastomotic angle and was concentrated in the heel and toe of the model.

Conclusions

The CFD modelling shows that increased acuity of anastomotic angles in ETS DIEP flaps is essential to minimize stagnation, recirculation, and WSS. Successful implementation of this recommendation may directly decrease the risk of flap failure from ischemia.

## Introduction

Microvascular anastomosis is a key facet of free-tissue transfers. The technique is commonly utilized with over 23,000 (16%) of the 137,808 breast reconstruction procedures conducted in the United States in 2020 being deep inferior epigastric artery perforator (DIEP) free-flap transfers [1].

The microvascular anastomoses within a DIEP flap can be classified as end-to-end (ETE) or end-to-side (ETS). The ETS technique is of particular importance because in many cases ETS may be preferable for its ability to preserve the internal mammary artery for use in future cardiac bypass surgery [2]. Further, many women who need breast reconstruction have a history of radiation therapy used to treat their breast cancer, resulting in damage to heart vessels which may lead to these patients being two times more likely to develop heart disease and become candidates for cardiac bypass surgery [3]. It is therefore important to understand how to enhance the outcomes of DIEP ETS anastomoses.

At present, no computational fluid dynamic (CFD) studies have been conducted in a DIEP ETS model. Existing cardiac and arteriovenous (AV)-shunt based CFD models do not translate as geometric dimensions and flow conditions differ between these models and DIEP ETS anastomoses [4].

This present study will create a CFD model for an ETS DIEP anastomosis to understand what angle(s) of vessel anastomosis can optimize blood flow and vessel wall forces. 

## Materials and methods

Geometric model

**Figure 1:**
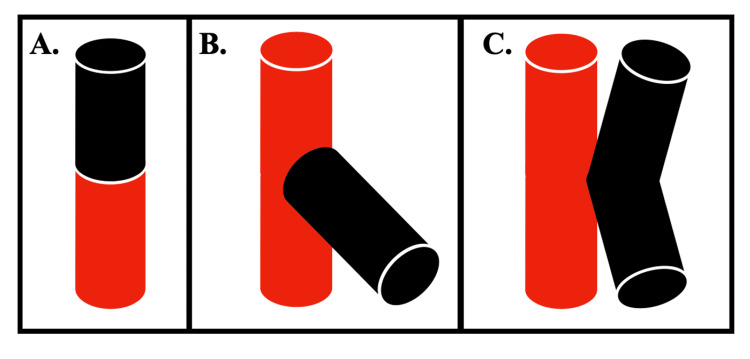
Types of microvascular anastomoses A. End-to-end (ETE) anastomosis; B. End-to-side (ETS) anastomosis; C. Side-to-side (STS) anastomosis

**Figure 2:**
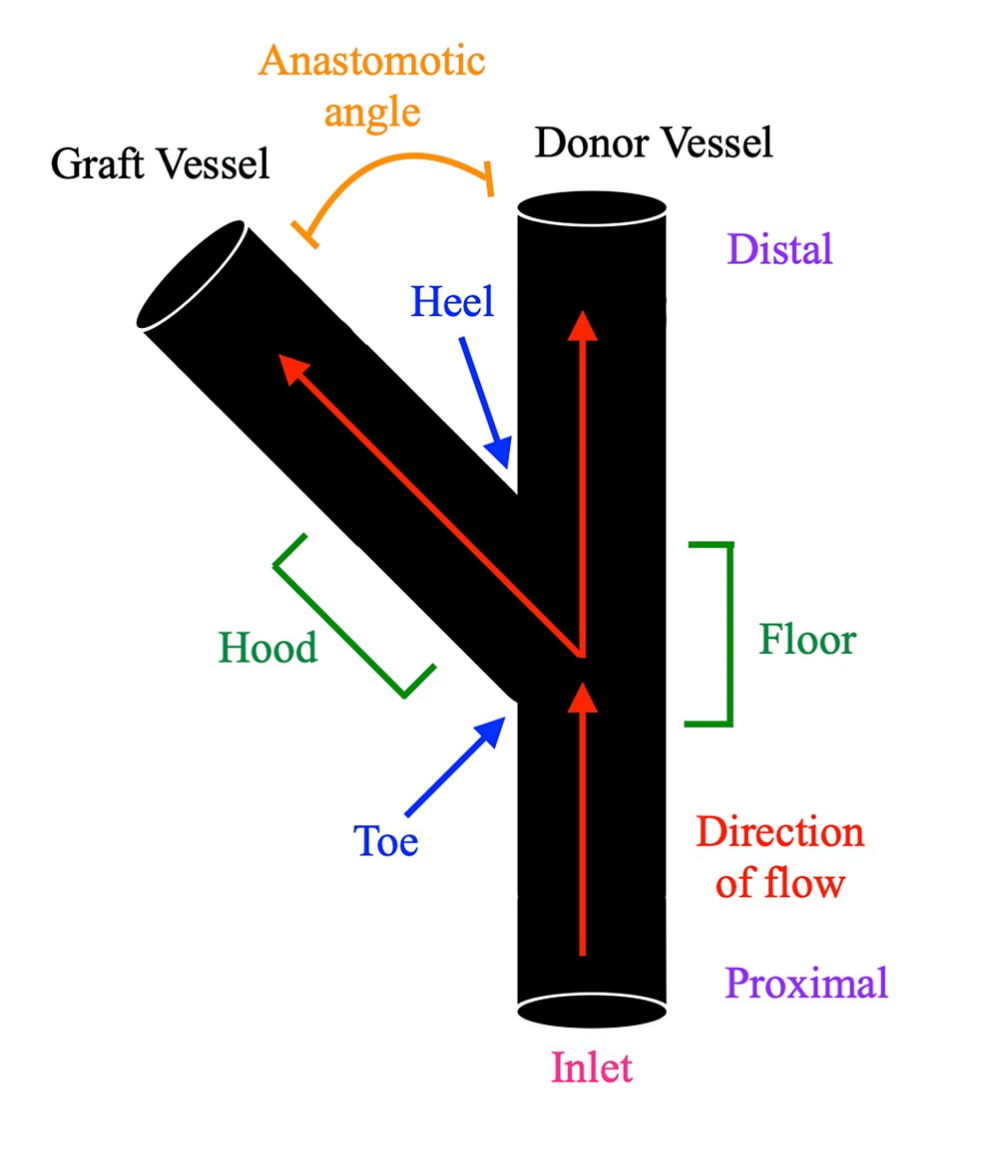
Anatomy of a microvascular anastomosis

A model was constructed to represent an ETS DIEP to internal mammary artery flap anastomosis (Figure 1 and Table 1). The deep inferior epigastric artery was defined as the graft vessel and the internal mammary artery as the donor vessel (Figure 2). Both vessels were modelled to have internal diameters of 2.3 mm with 200-micron wall thickness [5-7]. The length of the internal mammary and deep inferior epigastric arteries were defined as 184 mm [8] and 103 mm [6], respectively. The attachment point of the graft vessel to the donor vessel was defined as 100 mm distal to the inlet of the donor vessel. This attachment point was chosen to allow for sufficient vessel length to carry out fluid dynamic modelling on both the proximal and distal donor vessels. Anastomotic angles evaluated were 30-, 45-, 60-, 75-, and 90-degrees.

**Table 1:** Conditions of a geometric model for DIEP flap CFD simulation DIEP: Deep inferior epigastric artery perforator, CFD: Computational fluid dynamic

	Donor Artery (Internal Mammary) Dimensions	Graft Artery (Deep Inferior Epigastric) Dimensions
Internal Diameter (mm)	2.3	2.3
Wall Thickness (microns)	200	200
Length (mm)	184	103

Flow conditions

Blood flow was modelled as an incompressible Newtonian fluid with viscosity (η) of 3.5x10-3 Pa ⋅s and density (ρ) of 1060 kg/m3 [9,10]. Viscosity and density were assumed to be constant throughout the simulation process. Blood flow velocity (u) at the inlet was defined as 100 mm/s and the flow domain was pressurized to 100 mmHg, chosen to model mean arterial pressure (MAP) (Table 2).

**Table 2:** Newtonian flow conditions for DIEP flap CFD simulation

Condition	Parameter
Fluid viscosity (η)	3.5x10^-3^ Pa ⋅s
Fluid density (ρ)	1060 kg/m^3^
Fluid velocity at inlet (u)	100 mm/s
Pressure (p)	100 mmHg

Computational model

For CFD analysis OpenFOAM v9 was utilized to create a finite volume three-dimensional model [11]. Separate virtual meshes with an average of 1.1 million three-dimensional cells and four boundary layers were created for each anastomotic angle (30-, 45-, 60-, 75-, and 90-degrees). All simulations were run with a convergence tolerance of 10-4.

Wall shear stress (WSS) and fluid flow were visualized on each mesh. Fluid flow was visualized using both line integral convolution (LIC) and pure fluid velocity (PFV) techniques to allow for a better understanding of stasis and recirculation. Cross-sections of the PFV model were imported into the National Institutes of Health's (NIH) ImageJ [12] to calculate the percentage of blood in the graft vessels that were experiencing stagnation. Percentages were compared against anastomotic angles to construct a linear model.

## Results

**Figure 3:**
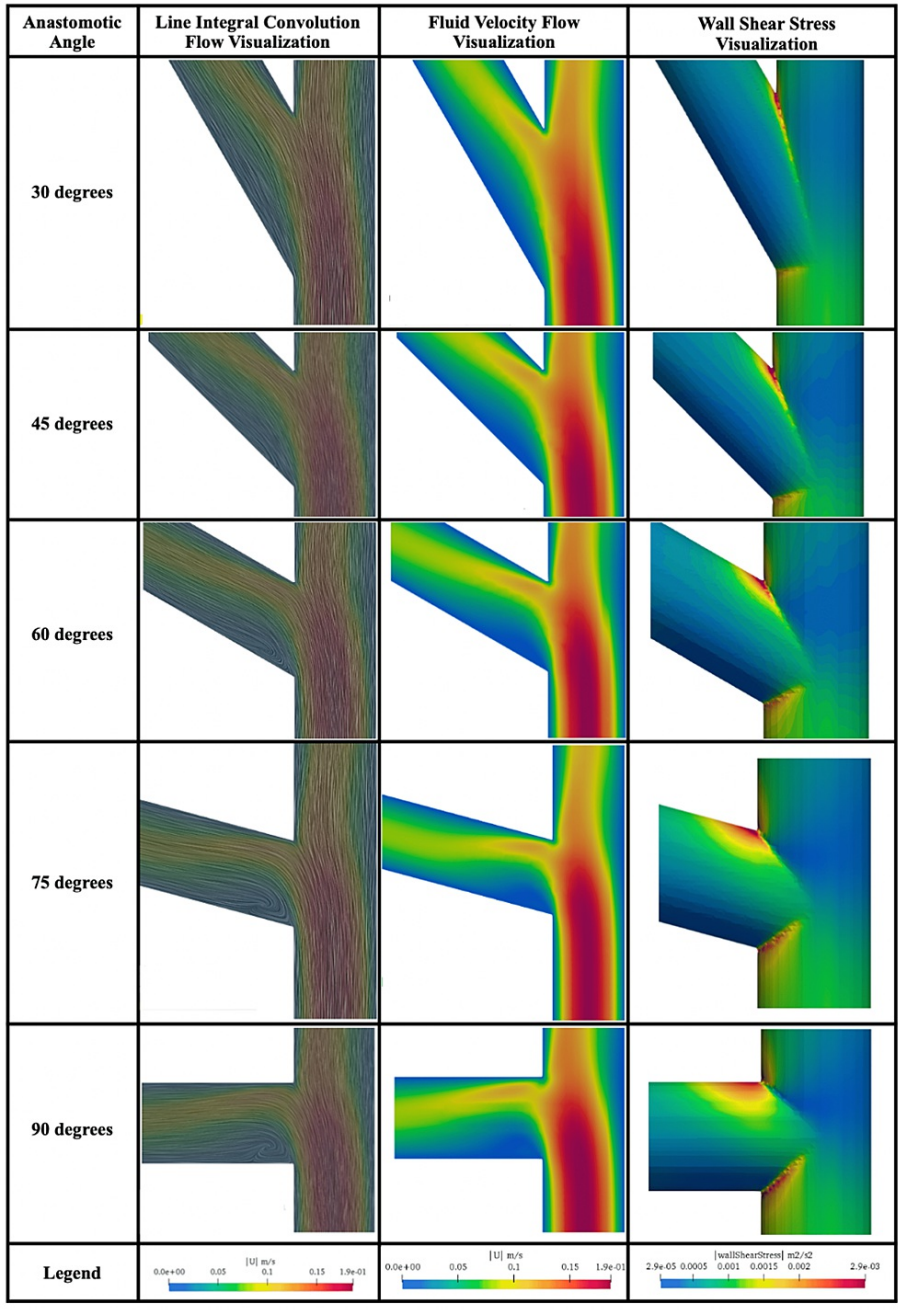
Computational fluid dynamic visualization of DIEP ETS anastomosis

Figure 3 illustrates the fluid flow (LIC and PFV) and WSS of all anastomotic models at convergence.

Line integral convolution

The LIC visualization for large angles revealed dense areas of recirculation in the toe of the bifurcation. The largest area of recirculation was observed with a 90-degree anastomotic model. The models approached laminar flow as the anastomotic angle decreased. Minimal to no recirculation was seen in both the 45- and 30-degree simulations. No recirculation was noted elsewhere in the LIC model with laminar flow notably present in the heel, hood, and floor in all simulations irrespective of anastomotic angle.

Pure fluid velocity

**Figure 4:**
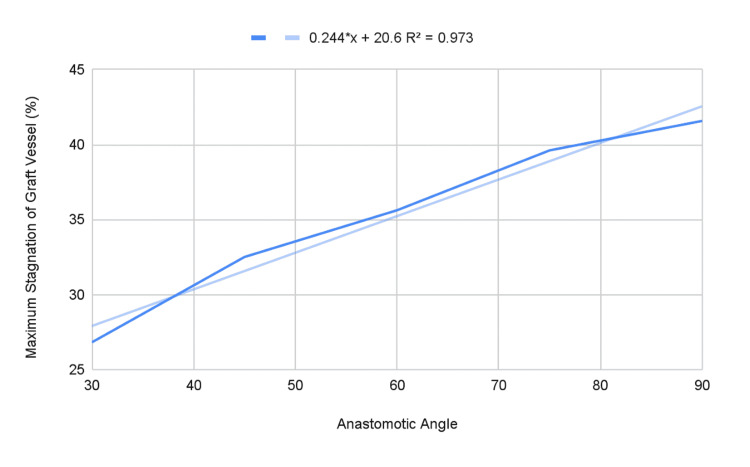
Maximum percentage of stagnation in a DIEP flap CFD model vs vessel angle

The PFV visualization revealed increased areas of stagnation in the toe of the graft vessel as the anastomotic angle increased. It also revealed that maximum cross-sectional stagnation in the graft vessel ranged from 26.8% at 30-degrees to 41.6% at 90-degrees (Table 3). The relationship between these variables was shown to be linear (R2 = 0.973) with the expression 0.244x + 20.6, where x is the anastomotic angle, predicting the maximum percentage of stagnation in a cross-section of the graft vessel (Figure 4).

**Table 3:** Maximum percentage of stagnation in a DIEP flap CFD model by vessel angle

Anastomotic Angle (degrees)	Maximum Stagnation (%)
30	26.82
45	32.51
60	35.63
75	39.62
90	41.58

Wall shear stress

Visualization of WSS revealed the area of abnormal vessel stress increased as the anastomotic angle increased. In the 30-degree simulation, WSS was concentrated at the heel of the model. As the anastomotic angle increased, WSS spread to include more of the heel and the donor vessel side of the toe. In all simulations, WSS was never concentrated on the hood or floor of the model.

## Discussion

This study is the first to use computational fluid dynamics to model a DIEP flap anastomosis using the end-to-side anastomotic technique. We make a strong case that an increasingly acute angle of anastomosis is associated with decreased stagnation, recirculation, and WSS.

Interestingly, recirculation was found to be nearly eliminated at both 45- and 30-degrees. This suggests the relationship between anastomotic angle and recirculation may be optimized at angles ≤45-degrees. Although no CFD models have been created for DIEP ETS flaps specifically, literature from other anastomotic CFD models supports our findings, suggesting a 45-degree angle will optimize flow [13,14].

Our identification of a linear relationship between anastomotic angle and maximum percentage of stagnation appears to be novel and is not reported elsewhere in the literature. Analysis of more acute angles than those included in our study is necessary to further validate this relationship.

Regarding WSS, our study identified stress was concentrated at the heel of the anastomotic bifurcation and progressively involved the toe as the vessel angle increased. A generic ex-vivo (non-CFD) model supports this finding, with photochromic tracers revealing increased angles are associated with increased shear stress at the toe [15]. However, the same model showed low shear stress was present at the heel of the anastomosis in all cases, irrespective of angle [15]. This variance from our study’s findings suggests stress at the heel is a unique facet of the geometry of DIEP flap anastomoses. Future analysis with new CFD or real-world models is necessary to further investigate this finding.

The cause of flap failure is often inadequate arterial perfusion due to the thrombogenic profile of anastomosed vessels creating insufficiencies in both outflow and inflow [16]. This is especially relevant for our DIEP ETS model because when ETS and ETE techniques were compared, within the context of breast reconstruction flaps specifically, ETS flaps were noted to have a higher mean ischemia time than ETE flaps [2,17]. While the ischemia time for ETS flaps remained within acceptable limits and incidence of thrombosis and fat necrosis were found to be similar between techniques, making considerations to minimize ischemia are essential for ensuring the continued success of ETS flaps [2,17,18].

Our findings directly address this issue of thrombosis related ischemia, suggesting an acute anastomotic angle optimizes the components of Virchow’s triad, namely minimizing endothelial injury (WSS) and stasis. Failure to consider this intraoperatively may lead to rapid thrombosis, ischemia, and acute flap failure [19]. The anastomotic angle should also be a consideration for long-term flap success with increased WSS and recirculation found to be associated with intimal hyperplasia and atherogenesis [19,20].

Limitations

The ETS DIEP flap dimensions can vary between patients. The measurements utilized for our geometric models are based on peer-reviewed averages for the vessels commonly utilized for this procedure, but a future study may be warranted that considers a range of measurements for each simulation. Furthermore, additional studies are underway to help characterize other flap/ETS permutations to permit greater clinical applicability. Additionally, blood was modelled as a Newtonian fluid with a defined MAP. Future analyses should evaluate blood with both Newtonian and non-Newtonian characteristics and consider the pulsatile conditions of human vasculature.

## Conclusions

Our study is the first to create a computational fluid dynamic model of a DIEP flap anastomosis using the end-to-side anastomotic technique. Although this technique is not the most commonly used anastomotic method for DIEP breast reconstruction, it is an option for some patients and surgeons. Further, this study outlines the physics behind ETS anastomoses, a mainstay of microsurgery which had not previously been studied.

We found increased acuity of microvascular anastomotic angles intraoperatively, in the context of ETS DIEP flaps specifically, is essential to minimize stagnation, recirculation, and wall shear stress. This consideration can directly decrease the risk of flap failure from thrombosis, intimal hyperplasia, atherogenesis, or other causes of acute and chronic ischemia. The success of this study serves as a jumping-off point for future research to be undertaken with varying pressures and vessel diameters to model other applications such as lower-extremity anastomoses. This analysis is currently underway.
